# Extreme Diversity of IgGs Against Histones, DNA, and Myelin Basic Protein in the Cerebrospinal Fluid and Blood of Patients with Multiple Sclerosis

**DOI:** 10.3390/biom10040630

**Published:** 2020-04-18

**Authors:** Irina A. Kostrikina, Valentina N. Buneva, Enrico Granieri, Georgy A. Nevinsky

**Affiliations:** 1 Institute of Chemical Biology and Fundamental Medicine, Russian Academy of Sciences, Siberian Division, 630090 Novosibirsk, Russia; irina@kostrikina.ru (I.A.K.); buneva@niboch.nsc.ru (V.N.B.); 2 Multiple Sclerosis Center, Department of Neurology, Ferrara University, 44121 Ferrara, Italy; gnr@unife.it

**Keywords:** multiple sclerosis, human cerebrospinal fluid, catalytic IgGs, DNase activity, protease activity, extreme diversity of IgGs against auto-antigens

## Abstract

It was recently shown that IgGs from sera of multiple sclerosis (MS) patients are active in the hydrolysis of DNA and myelin basic protein (MBP). We first analyzed the relative concentration of antibodies against five histones (H1, H2a, H2b, H3, and H4) in the cerebrospinal fluid (CSF) and serum of patients with MS. The relative concentrations of blood and CSF IgGs against histones and their activity in the hydrolysis of five histones varied greatly from patient to patient. However, all 28 IgG preparations were hydrolyzed from one to five histones. Relative activities and correlation coefficients among the activities of IgGs from serum and CSF in the hydrolysis of five histones (H1, H2a, H2b, H3, and H4), DNA, and MBP were calculated. It was shown that auto-IgGs from CSF and sera of MS patients are extremely heterogeneous in their affinity to histones, MBP, and DNA. The heterogeneity of IgG-abzymes hydrolyzing DNA, MBP, and histones from CSF and sera was also demonstrated using their isoelectrofocusing. The isofocusing profiles DNase, MBP-, and histone-hydrolyzing activities of IgGs may be very different for various individuals, but the total IgG subfractions with all their activities are distributed from pH 3 to 10.

## 1. Introduction

Multiple sclerosis (MS) presenting a serious medical and social problem is a chronic demyelinating pathology of the central nervous system. Its etiology still remains unclear, and the most valid pathogenesis theory assigns the main role in the destruction of the axons myelin-proteolipid shell to inflammation related with autoimmune-mediated reactions ([1], and refs therein). In an enhanced synthesis of IgGs, their free light chains can be observed in MS patients [1]. However, the cloning IgG repertoire from active plaques and periplaque regions of the brain and recovered from the cerebrospinal fluid B-cells of MS patients’ spine was carried out [2]. High affinity anti-DNA IgGs were revealed as a major component of the intrathecal IgG response in MS patients. Anti-DNA-specific Abs from MS and systemic lupus erythematosus (SLE) patients interacted efficiently with the surface of neuronal cells and oligodendrocytes. The results indicated that auto-Abs against DNA may stimulate important neuropathologic mechanisms not only in SLE but also in MS patients [2].

It is believed that several pathogens may be associated with the development of MS including bacteria (such as *Chlamydia pneumoniae*, *Mycoplasma pneumoniae*, and *Staphylococcus*) producing different superantigens, as well as viruses (human herpesvirus, Epstein–Barr virus, and human endogenous retroviruses) (for review see [3,4] and refs therein). It is known that after an infection with viruses and bacteria, at first there is an accumulation of antibodies against their components, which may have structural similarities with the components of blood and human cells [3,4]. Then, due to the mimicry of certain proteins of viruses and bacteria with those of humans, epitope spreading and bystander activation, a failure of the immune system can occur leading to the generation of Abs against its own components of the human body and, as a result, to the development of MS.

One of the features of MS is the appearance of oligoclonal IgGs in the bone marrow CSF, revealing themselves as oligoclonal bands (OCBs) after CSF proteins isofocusing [5,6,7]. Nevertheless, OCBs were found in the CSF of patients with MS who were sick and non-sick with various bacterial and viral diseases. In most cases, after detection using ELISA in the CSF of patients with MS antibodies against components of viruses or bacteria, there are no data whether these Abs contain oligoclonal IgGs against viral or bacterial components [3]. By using isoelectrofocusing with following antigen-specific immunoblotting, specific OCBs in CSF of some MS patients were revealed definitely against the only components of Epstein–Barr virus [8] and Chlamydia [9]. However, it is still unknown which human auto-antigens oligoclonal antibodies may be produced in MS patients.

Artificial abzymes (Abzs, catalytic antibodies to transition state analogues of chemical reactions) and natural Abzs have attracted much interest in the last years (reviewed in [10,11,12,13,14,15]). Similar to artificial Abzs against the analogs of transition states, natural abzymes can be produced against directly enzyme substrates acting as haptens simulating the transition states of chemical reactions. Abzs can also be anti-idiotypic antibodies against the active centers of various enzymes. Natural abzymes hydrolyzing oligopeptides, proteins, DNA, RNA, nucleotides, and polysaccharides are present in the serum of patients with autoimmune and viral diseases (e.g., SLE, MS, Hashimoto thyroiditis, polyarthritis, tick-borne encephalitis, and HIV-infected patients) [10,11,12,13,14,15]. Healthy humans do not usually develop Abzs with detectable enzymatic activities; their levels are usually on the borderline of the sensitivity of the detection methods [10,11,12,13,14,15]. Nevertheless, germline Abs of healthy people can possess high level promiscuous, amyloid-, or superantigen-directed activities [16].

The statistically significant appearance of Abzs is detected at the earliest stages of various autoimmune diseases (AIDs), when changes in antibody titers to specific antigens of various diseases, such as DNA (SLE, MS, etc.), myelin basic protein (MS, SLE, etc.), thyroglobulin (Hashimoto thyroiditis), and other proteins, still correspond to the range changes in the titers of these antibodies in healthy donors [10,11,12,13,14,15]. According to modern data, the presence of abzymes in the blood is a clear sign of the beginning and progress of autoimmune processes in human and mammals.

It was shown that myelin basic protein (MBP)-, DNA-, and oligosaccharide-hydrolyzing activities are intrinsic property of IgGs, IgMs, and IgAs from sera of MS and SLE patients [17,18,19,20,21,22]. The anti-MBP Abzs with protease activity can attack MBP of the myelin-proteolipid sheath of axons and may therefore play an important negative role in MS pathogenesis [14]. DNase Abzs are cytotoxic, cause fragmentation of nuclear DNA and induce cell death by apoptosis [15]. We have recently shown that the relative specific enzymatic activities of Abs from the CSFs of patients with MS in the hydrolysis of DNA, MBP, and oligosaccharides are about 30–60-fold higher than from the blood serum of these patients [23,24,25].

Histones in addition to various intranuclear cell functions act as damage-molecules in the extracellular space [26]. The blood-level increase in histones is associated with multiple adverse pathophysiological processes including the progression in various AIDs and inflammatory diseases [26]. It was recently shown that in contract to healthy humans, 100% of IgGs purified from the sera of 32 HIV-infected patients efficiently hydrolyze from one to five human histones (H1, H2a, H2b, H3, and H4) [27]. In addition, the blood and brain of experimental autoimmune encephalomyelitis (EAE)-prone C57BL/6 mice contain auto-Abs against histones effectively hydrolyzing these proteins [28]. Many anti-DNA Abs are directed against nucleosomal histone-DNA complexes appearing during cell apoptosis [29]. Therefore, not only harmful abzymes with DNase activity, but also Abs hydrolyzing histones may be important for the development of MS.

Recently it was shown that the blood of patients with MS contains antibodies specifically hydrolyzing histones [30]. It is still unknown if the cerebrospinal fluid of patients with MS contain autoantibodies and abzymes against histones. Along with DNA and MBP, histones were considered by us as potential antigens for which auto-IgGs can be accumulated in the CSFs and sera of patients with MS. In addition, in literature there is no data on the possible diversity of autoantibodies and abzymes in the CSF of patients with any diseases, including MS. In this work, for the first time, we analyzed the diversity of auto-antibodies from the cerebrospinal fluid and blood of patients with MS by their affinity for DNA, MBP, and histones, as well as the distribution of IgGs hydrolyzing these substrates at isoelectric points (*pI*) after antibody isoelectric focusing. It has been shown that in CSFs and sera, IgGs are characterized by exceptional diversity in affinity for the antigens and the relative catalytic activities.

## 2. Materials and Methods

### 2.1. Patients, Donors, and Chemicals

Most chemicals, proteins, the Superdex 200 HR 10/30 column, and protein G-Sepharose were from Sigma (St. Louis, MO, USA) or GE Healthcare (GE Healthcare, New York, USA). Twenty-eight consecutive MS patients (20 women and 8 men; mean age = 38.7 ± 12.3 years), satisfying according to the classification of McDonald [31], the criteria for definite MS and admitted to the Multiple Sclerosis Center of the Ferrara University during the period from January 2014 to October 2017, were selected for the study. The blood and CSF sampling protocols conformed to the local hospital human ethics committee guidelines (Multiple Sclerosis Center, University of Ferrara, Italy) in accordance with Helsinki ethics committee guidelines. Informed consents were given by all patients prior to inclusion. The design of the study was supported by the Regional Committee on Medical Ethics in Research.

Disease severity of the MS patients analyzed was scored using Kurtzke’s Expanded Disability Status Scale (EDSS) [32] (range from 0.0 to 6.5, average mean 2.6 ± 1.3) at the time of sample collection. At entry all patients had no fever or symptoms of acute infections. In addition, none of the patients at the time of sample collection had received any anti-disease therapies during the six months before the study. More detailed data are given in the Supplementary Table S1.

### 2.2. Sample Preparation

Samples of CSF and serum were collected using sterile conditions and preserved in aliquots at −80 °C until assay. CSF samples was taken by atraumatic lumbar puncture and then free of cells preparations were obtained using centrifugation at 20 °C for 15 min at 12,000 rpm using an Eppendorf centrifuge (New York, USA). Simultaneously blood preparations with CSF samples were obtained by puncture of the anterolateral vein and then they were centrifuged for the removing of cells.

### 2.3. Purification of IgGs

Electrophoretically homogeneous IgGs were obtained using first affinity chromatography of the serum and CSF proteins on protein G-Sepharose and then by FPLC gel filtration as in [17,18,19]. The content of IgGs in serum and CSF of different patients as well as their concentration after their purification was estimated by standard immunochemical nephelometry according to [7,8,9,33,34].

### 2.4. DNase Activity Assay

DNase activity of IgG preparations was analyzed as in [21,33]. The reaction mixtures (20 µL) containing 20 µg/mL supercoiled (sc) DNA pBluescript, 1 mM EDTA, 3–5 mM MgCl_2_, 20 mM Tris-HCl (pH 7.5), and 0.003–0.2 mg/mL IgGs were incubated for 0.5–4h (standard time, 2 h) at 37 °C. The products of DNA hydrolysis were analyzed using electrophoresis in 1% agarose gel. All initial rates of the reaction were estimated using the linear regions of the time courses and IgG concentration (20–40% of scDNA hydrolysis). The ethidium bromide-stained gels were analyzed using a Sony DSC-F717 camera (Sony Center, Berlin, Germany) and ImageQuant v5.2 (Molecular Dynamics, New York, NY, USA). The activities of IgGs were found from a decrease in scDNA corrected for the distribution of DNA between bands of initial and the relaxed form of DNA in the control (incubation of scDNA in the absence of Abs). A complete transition of the scDNA to its relaxed form after 1 h of incubation was taken for 100% activity. Finally, relative DNase activity was calculated as pmole DNA/1 mg of IgGs/1 h. DNase activity was also determined using this approach after isoelectrofocusing of IgGs.

### 2.5. Protease Activity Assay

The reaction mixtures (10–30 μL) for analysis of MBP- and histone-hydrolyzing activities of IgGs contained 20 mM Tris-HCl (pH 7.5), 0.7–1.0 mg/mL MBP or mixture of histones, and 0.01–0.2 mg/mL of IgGs. They were incubated for 1–21 h at 37 °C. The cleavage products of the proteins were analyzed after SDS-PAGE using 12% or 4–15% gradient gels; proteins were stained using Coomassie R250. The gels were scanned and quantified by GelPro v3.1 software (Media Cybernetics, Silver Spring, MD, USA). The relative activities (RAs) of different IgGs were determined from a decrease in the percentage of non-hydrolyzed proteins converted to their different shorted forms, taking into account percentage of the hydrolysis of control MBP or histones without Abs. All initial rates (% of the hydrolysis) were estimated using the conditions of the pseudo-first order reaction within the linear regions of the IgG concentrations and time course (20–40% hydrolysis of the proteins). Finally, relative protease activity was recalculated as nmole MBP/1 mg of IgGs/1 h. Protease activity of IgGs was also determined in the same way after isoelectrofocusing of IgGs.

### 2.6. SDS-PAGE Assay of Ab Proteolytic Activity

SDS-PAGE analysis of IgGs was performed in 4–15% gradient gels under nonreducing conditions (0.1% SDS) and proteins were detected by silver staining as in [17,18,19,20,21,22,23,24]. For the analysis of catalytic activities IgGs (10–15 µg) before electrophoresis were preincubated at 30 °C for 20 min in 50 mM Tris-HCl, pH 7.5, containing 1% SDS, and 10% glycerol. To restore protease activity after SDS-PAGE, SDS was removed by incubation of the gels at 22 °C for 2 h with 50 mM Tris-HCl containing 4 M urea, and the gel was washed seven times with water. The gel cross-sections of longitudinal slices (3–4-mm) were cut out and incubated for five days at 4 °C with 100 μL of 25 mM Tris-HCl, pH 7.5, containing 5.0 mM MgCl_2_ and 1.0 mM EDTA to allow IgGs refolding and eluting from the gel. The eluates were used as described above for the activity assay. Parallel gel longitudinal slices were used to detect the position of IgGs in the gel by Coomassie staining.

### 2.7. Isoelectrofocusing of IgGs

The separation of purified IgGs by isoelectrofocusing was carried out using long 18 cm gels from Protein isoelectrophocusing (IEF) Cell (Bio-Rad, New York, USA). IgGs in solubilizing buffer containing 8 M urea, 2% Nonidet P-40, 0.2% ampholine pH 3–10, and 50 mM dithiothreitol were added in the IEF cell (0.32 mL) according to manufacturer’s procedure. The special linear strips (pH 3–10, 18 cm; Bio-Rad, USA) were used in the pockets of which mineral oil was added. The strips were passively dehydrated for 1 h, and then actively for 12 h. In the pockets of the gels 0.3 solutions of purified IgGs (1 mg/mL) were added. Isoelectrofocusing was carried out for 15 min at 250 V, then for 7 h at 10^4^ V. Then the IEF gels were cut into vertical streaks. To detect the position of proteins some IEF streaks were incubated for 30 min in buffer containing 0.38 M Tris-HCI (pH 8.8), 6 M urea, 20% glycerol, 2% SDS, and 0.001% Coomassie blue. The parallel streaks were washed twice with water and cut into 0.45–0.5 cm cross fragments carefully disrupted and placed in tubes containing 50 µL of 50 mM Tris-HCl buffer (pH 7.5). The tubes were incubated for 1 day at 4 °C to allow IgGs refolding and eluting from the gel pieces. The eluates were removed from the gels by centrifugation and used for assay of DNA, MBP, and histone hydrolysis as described above.

### 2.8. Affinity Chromatography of IgGs on Different Sorbents

IgGs from CSF of MS patients were chromatographed on Sepharose bearing immobilized histones, human MBP, and DNA-cellulose according to [19,20,21,35,36] as described earlier for analysis of Abs from sera of patients with different AIDs. The columns were equilibrated with Tris-HCl buffer (50 mM, pH 7.5) containing 50 mM NaCl; the IgG preparations were applied, and the columns were washed with the same buffer to obtain zero optical density. IgGs were eluted from these affinity sorbents sequentially with the buffer containing 3 M NaCl, 3 M MgCl_2_, and finally acidic Tris-glycine buffer (pH 2.6). Fractions after all chromatographies were dialyzed against 50 mM Tris-HCl (pH 7.5) containing 50 mM NaCl and used for the estimation of IgG activity in the hydrolysis of DNA, MBP, and histones.

### 2.9. Statistical Analysis

The results are reported as average mean ± S.E. of at least three independent experiments for each sample analyzed. Errors in the values for individual IgGs were within 5–15%. To check for a normality of the values distribution the criterion of Shapiro–Wilk’s W Test was used. Most of the sample sets did not meet the normal Gaussian distribution. Therefore, the nonparametric ranking method of Spearman was mainly used for the correlation analysis. In the case where the data obeyed to the normal distribution law, the Pearson parametric method was used. To evaluate the differences between the samples, the Mann–Whitney U test was used; *p* < 0.05 was considered statistically significant. The median (M) and interquartile ranges (IQR) were estimated.

## 3. Results

### 3.1. Anti-Histones Abs in CSF and Sera of MS Patients

In this paper, we first analyzed concentrations of total IgGs in the CSF and sera samples of 28 patients with MS. The main characteristics of these patients are listed in Supplementary Table S1.

Then, we estimated the relative concentrations of Abs against histones using an equimolar mixture of five histones (H4, H1, H2a, H2b, and H3) in the CSF and serum preparations. Taking into account the large difference in the concentration of Abs against histones in the cerebrospinal fluid and in the serum, the initial samples of these fluids were diluted 10 and 100 times, respectively for ELISA. Then, the obtained A_450_ values were recalculated to the same 100-fold dilution and expressed in standard ME units. For 28 CSF preparations, these values varied from 0.01 to 0.042 ME (average value 0.02 ± 0.01 ME), and in sera from 0.9 and to 4.2 ME (average value 2.5 ± 0.87 ME). The average concentration of auto-Abs against histones in sera is ~125-fold higher than that in CSF preparations.

### 3.2. DNA and MBP-Hydrolyzing Activity of the Serum and CSF IgGs

It was previously shown that the CSFs of fifteen MS patients contain IgGs effectively hydrolyzing DNA, MBP, and oligosaccharides [23,24,25]. In addition, the specific activity of IgGs from CSF in hydrolysis of DNA, MBP, and oligosaccharides is about 30–60 times higher than that for Abs from the blood sera of the same patients [23,24,25]. As noted above, histones themselves can play an important negative role in the development of various autoimmune diseases [26].

For comparison, in this article we have analyzed DNA-, MBP-hydrolyzing activity of a new set of 28 MS patients (Table 1). It was shown that DNase activity of IgGs from CSFs is 45.0-fold higher than that from sera of the same 28 patients and the correlation coefficient (CC) between these values is +0.26 (*p* < 0.05). These data are in agreement with a previously published estimation for another set of 15 patients: CSF IgGs are 48.5-fold more active than from sera and CC = +0.26 (*p* < 0.05) [23]. MBP-hydrolyzing activity from CSF of 28 new patients was 55.6-fold higher than from sera and CC between this values was +0.45 (*p* < 0.05) (Table 1), while in the previous article [24] these values were 58.6-fold and CC = +0.43, respectively. Thus, two sets of MS patients demonstrate similar data on relative activity and CCs for IgGs from CSF and blood of patients with MS. It was shown that the CC between the activities of CSF IgGs in the hydrolysis of DNA and MBP is equal to −0.07, and from serum, +0.17. The analysis of IgGs against histones and a comparison of the relative activity of abzymes in the hydrolysis of histones with the RAs in splitting of DNA and MBP was of particular interest.

### 3.3. Evidence of Histone-Hydrolyzing Activity Directly to Antibodies

**Figure 1:**
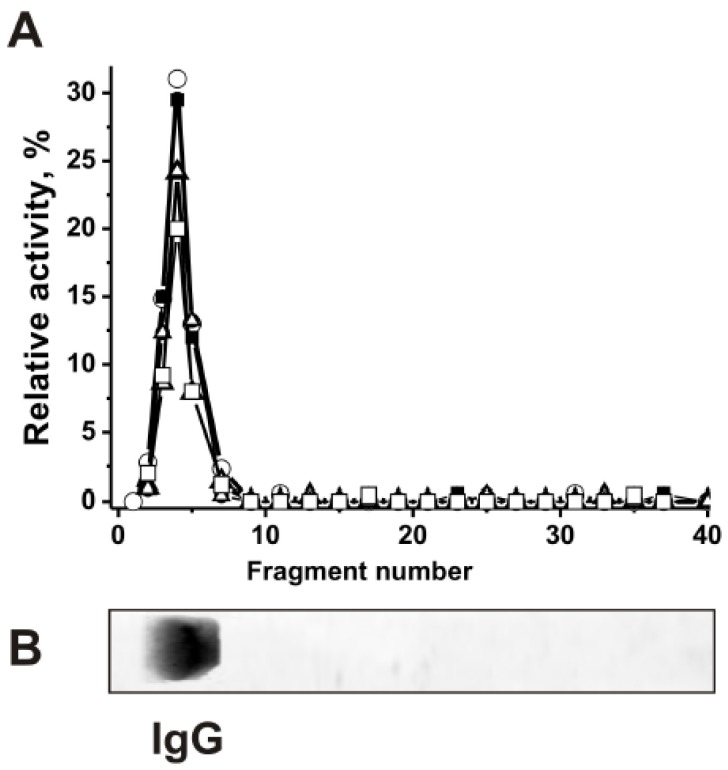
SDS-PAGE analysis of CSF IgG_mix_ (mixture of ten IgGs) histones-hydrolyzing activities (**A**). After non-reducing (in the absence and without preincubation with DTT) SDS-PAGE of csf-IgG_mix_ in 4–15% gradient gel, the gel was incubated using special conditions for renaturation of IgGs. The relative activities (RAs) in the hydrolysis of H1 (○), H2a (□), H2b (■), H3 (∆), and H4 (●) were revealed using the extracts of many 3–4-mm fragments of one longitudinal slice of the gel (**A**). The RAs of csf-IgG_mix_ corresponding to the complete hydrolysis of all histones after 20 h of the incubation with 12 μL of extracts was taken for 100%. The second longitudinal control slice of the same gel was stained with colloidal silver (**B**). The error in the initial rate calculation from three independent experiments did not exceed 7–15% (**A**).

It is known that anti-DNA Abs are mostly directed against histone-DNA complexes appearing in blood during cell apoptosis [29]. IgGs purified from blood sera of HIV-infected [27] and MS [30] patients as well as EAE-prone mice [28] efficiently hydrolyze from one to five human histones (H1, H2a, H2b, H3, and H4). However, proteolytic activity auto-IgGs against histones from CSFs of patients with MS in the hydrolysis of five histones has not been previously studied. Here it was first shown that hydrolysis of five proteins (H1, H2a, H2b, H3, and H4) is intrinsic property of IgGs from CSFs and sera of MS patients. The mixture of equal amounts of 17 IgGs from the sera (s-IgG_mix_) and CSFs (csf-IgG_mix_) with maximal proteolytic activities was prepared. Then both IgG_mix_ were separated by SDS-PAGE and their protease activities were detected after proteins extraction from the separated gel slices. Figure 1 demonstrates the data for csf-IgG_mix_. SDS-PAGE revealed proteolytic activity in the hydrolysis of all five histones (H1, H2a, H2b, H3, and H4) only in the band corresponding to intact csf-IgG_mix_ preparation (Figure 1). Similar results were obtained for s-IgG_mix_ from sera. Since SDS can dissociate any protein complexes, and the low molecular mass of canonical proteases (25.7–28.5 kDa) electrophoretic mobility cannot coincide with that of intact IgGs (150 kDa), the detection of protease activity in the gel region corresponding only to intact IgG_mix_ together with the absence of any other bands of proteins and any additional peaks of the activity (Figure 1), ensures direct evidence that the IgG_mix_ preparations possess histones-hydrolyzing activities. Thus, we present a first evidence demonstrating that IgGs from CSF of MS patients efficiently hydrolyze five histones.

### 3.4. Determination of Relative Histone-Hydrolyzing Activity of the Serum and CSF IgGs

**Figure 2:**
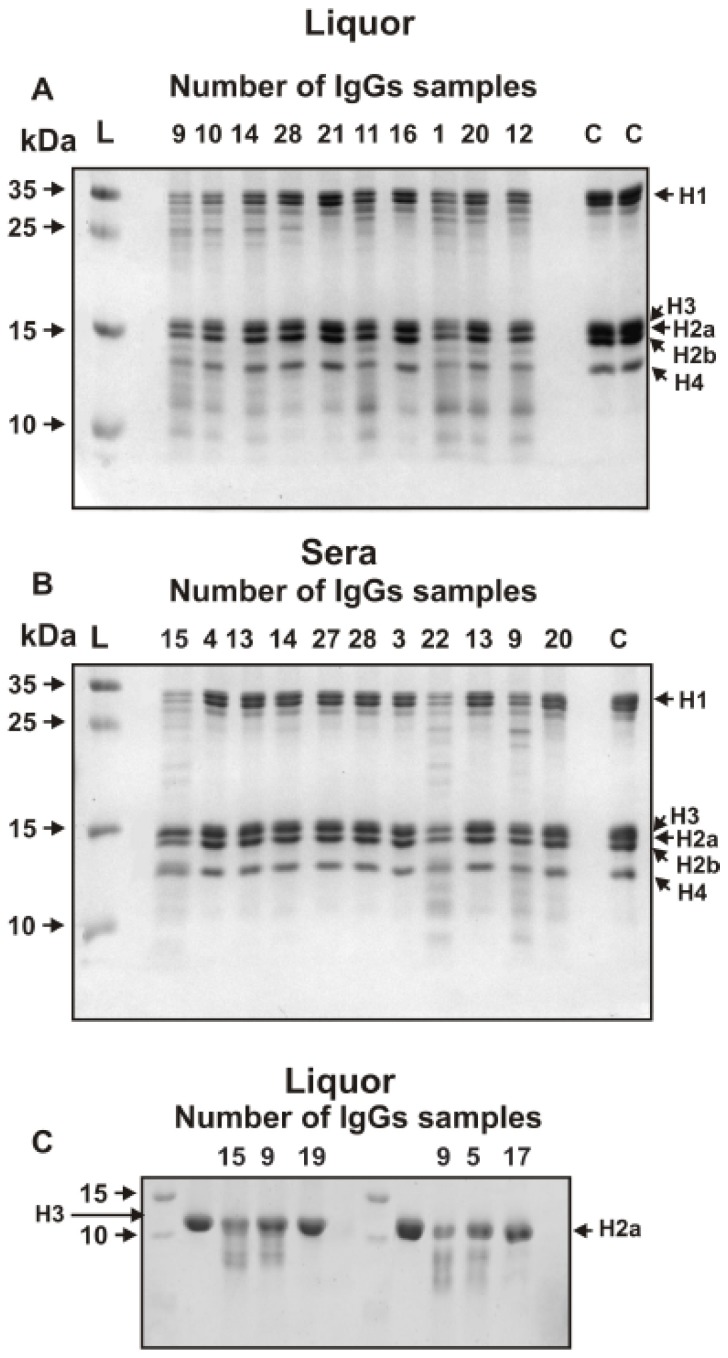
SDS-PAGE analysis of five histones hydrolysis by several individual IgGs from CSF (**A**), sera (**B**), and homogeneous H3 and H2a histones (**C**). Before electrophoresis the reaction mixtures containing 1 mg equimolar mixture of five histones (**A**,**B**) or homogeneous H3 and H2a (**C**) were incubated for 17 h at 37 °C in the presence of 0.005–0.01 mg/mL IgGs from CSFs or 0.1–0.2 mg/mL Abs from sera. Lanes C correspond the incubation of histones without Abs; the arrows corresponding lane L indicate the positions of molecular mass markers.

Hereafter the estimation of relative activity of electrophoretically homogeneous MS IgGs from CSF and sera in the hydrolysis of five histones was carried out. Figure 2 shows first data on the hydrolysis of the histones by IgGs from CSFs (Figure 2A) and sera (Figure 2B) by several IgG preparations using equimolar mixture of five histones. Unfortunately, the electrophoretic mobility of H3 and H2a histones almost coincides. From such data it was possible to evaluate the relative efficiency of the hydrolysis of only H1, H2b, and H4 histones and average values of hydrolysis of sum of H3 and H2a histones. Therefore, the data on the hydrolysis of H3 and H2a were further analyzed additionally by the hydrolysis of homogeneous preparations of these histones (for example, Figure 2C). Then, the average hydrolysis values (%) of different histones were estimated, based on data from three to five independent experiments.

The activity of IgG antibodies in the hydrolysis of five histones from the CSF was significantly higher than that from blood sera. Therefore, the activity of IgGs from the CSF was estimated using the concentration of Abs in the reaction mixtures from 0.005 to 0.01 mg while from sera from 0.1 to 0.3 mg/mL. Then the relative activity of all preparations was recalculated (%) to the standard conditions corresponding to 0.1 mg/mL IgGs/15 h. These data obtained are given in Table 2. One can see that the RA in the hydrolysis of five histones is significantly varying depending of patients and histone analyzed. Not all data of different sets corresponded to the Gaussian distribution; therefore, for all data, the median (M) and interquartile ranges (IQRs) were calculated. RA in the hydrolysis of H1 by IgGs from CSFs varies from 0 to 671% (average value 121.9 ± 148.6%; M = 98.5; IQR = 154), while from sera of the same patients from 0 to 24% (average value 6.9 ± 7.6; M = 4.5; IQR = 12.8); the ratio of these average values is 17.7, while CC = −0.1. The RAs of the hydrolysis of H2a by CSF IgGs varied in range 0–440% (average value 205 ± 107%; M = 196; IQR = 159) that 16.8-fold higher than for Abs from sera: the range 0–29% (average value 12.3 ± 10.3%; M = 13.2; IQR = 20.9); CC= −0.1 (Table 2). All 28 preparations hydrolyze H2b; RAs varied from 10 to 120% (average value is 40.3 ± 39.6%; M = 14.5; IQR = 66.0), when only 7 of 28 sera IgGs were active in the hydrolysis of H2b: the range of RAs is 0–13.2% (average value is 2.2 ± 4.3%; M = 0; IQR = 12.5). CSF IgGs are ~18.3 times more active then sera Abs in the hydrolysis of H2b; CC = +0.23 (Table 2).

Only one IgG preparation of CSFs was inactive in the hydrolysis of H3; the RAs were varying from 0 to 376% (average value 136.5 ± 98.3%; M = 120; IQR = 125), while only 14 of 28 preparations of sera hydrolyze H3: 0–21% (average value 5.3 ± 6.4%; M = 3.1; IQR = 10.0). The difference in these average RAs of CSF and sera IgGs is 25.8; CC= +0.05.

IgGs from CSF demonstrated relatively high activity in the hydrolysis of H4 from 0 to 524% (average value 147 ± 143%; M = 110; IQR = 162). A total of 11 of 28 IgGs from sera were inactive in the hydrolysis of H4; their RAs varied from 0 to 21%: average value 5.4 ± 6.0% (M = 4.9; IQR = 7.1). RA of sera IgGs was 27.2-fold lower than that for CSF IgGs; CC= +0.43. Overall, the average activity of IgGs from CSF in the hydrolysis of all five histones decreases in the following order: H2a > H4 > H1 > H3 > H2b, while for IgGs from sera H2a > H1> H3 ≈ H4 > H2b (Table 2). The CCs between the activities of IgGs from the CSFs and blood vary from negative (−0.1) to positive (+0.43). In overall, the average activity of IgGs from CSFs in the hydrolysis of five histones is approximately 21.2 ± 4.9 times higher than from sera. It is interesting that the difference between IgGs from CSFs and sera in the hydrolysis of DNA (45-fold) and MBP (55.6-fold; Table 1) is significantly higher than that for the histones (Table 2). Nevertheless, all IgG preparations from CSFs and sera of patients with MS hydrolyze from 1 to 5 histones (Table 2).

We have analyzed CCs between average hydrolysis of DNA and five histones by IgGs from CSFs: H1 (−0.22), H2a (−0.02), H2b (−0.16), H3 (−0.2), H4 (+0.05), and DNase and histone-hydrolyzing activity from sera: H1 (+0.05), H2a (+0.03), H2b (−0.3), H3 (+0.56), H4 (+0.05). The CCs between average activity of CSF IgGs in the hydrolysis of MBP and five histones were also estimated: H1 (−0.14), H2a (+0.28), H2b (−0.46), H3 (+0.18), H4 (+0.16), as well as for sera IgGs: H1 (−0.13), H2a (−0.15), H2b (−0.18), H3 (+0.32), H4 (+0.19). One can see that CCs are very low and vary from negative to positive values. In addition, some CCs for IgGs of CSF and sera have directly opposite meanings. For instance, CC for DNA- and H3-hydrolyzing activity of IgGs from CSFs is -0.2, when from sera IgGs, it is +0.56. Such very different CCs for various activities in MS patients may be a consequence of the very great and specific heterogeneity of abzymes in each patient.

### 3.5. Heterogeneity of CSF IgGs Analyzed by Affinity Chromatographies

**Figure 3:**
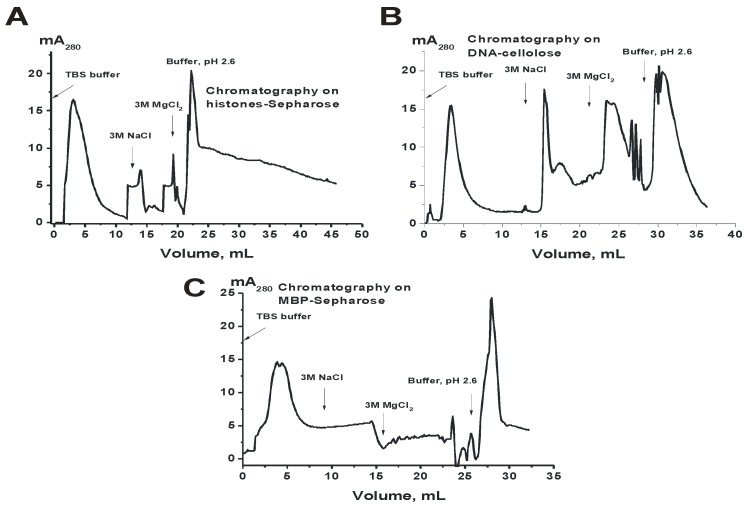
Profiles of the affinity chromatographies of CSF IgG_mix_ on histones-Sepharose (**A**), DNA-cellulose (**B**), and myelin basic protein (MBP)-Sepharose (**C**) demonstrating extraordinary heterogeneity of csf-IgG_mix_ in their affinity to histones, DNA, and MBP.

The extreme diversity of IgGs in the affinity to DNA, MBP, and other proteins from the sera of patients with MS, SLE, and autoimmune prone MRL-lpr/lpr mice was observed [11,12,13,14,15]. It was shown that sera Abs against these ligands can be separated into many fractions by chromatography on DNA-cellulose and protein-Sepharoses [19,20,21,35,36]. In this work we analyzed for the first time the common level of possible heterogeneity of IgGs from CSFs and sera of MS patients. Since there were relatively small amounts of CSFs IgGs, for the analysis of possible common heterogeneity of IgGs in affinity to histones, DNA, and MBP csf-IgG_mix_ was used. Figure 3 demonstrates the distribution of csf-IgG_mix_ (equimolar mixture of IgGs from CSFs of 28 patients) over the entire profile during affinity chromatography on histone-Sepharose, DNA-cellulose and MBP-Sepharose. Similar results were obtained for s-IgG_mix_ corresponding to 28 preparations from sera of the same patients. It means that CSF and sera IgGs of MS patients are extremely heterogynous in their affinity for DNA, MBP, and histones.

### 3.6. Heterogeneity of CSF and Sera Abzymes Analyzed by Isofocusing

**Figure 4:**
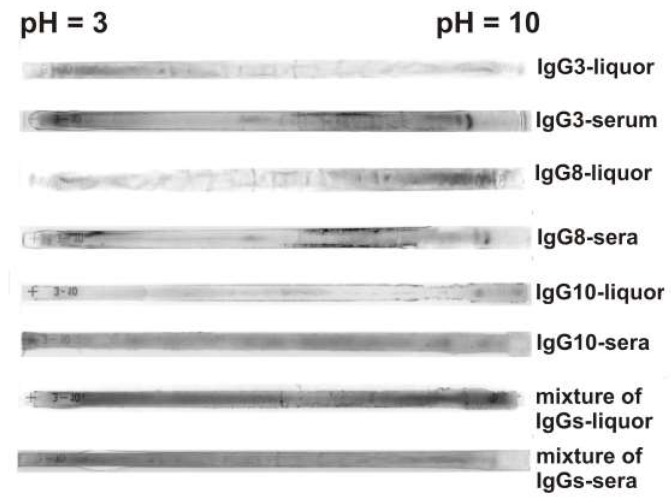
Typical examples of the patterns of the distribution of total polyclonal IgGs from CSFs and sera after their isofocusing. Longitudinal strips of the gels after isoelectric focusing were painted with Coomassie blue. The numbers of IgG preparations are shown in the figure.

The heterogeneity of abzymes hydrolyzing DNA, MBP, and histones from sera and CSFs of MS patients was also demonstrated by isofocusing. For a more efficient separation of all subfractions of purified IgGs from CSFs and sera, isoelectric focusing of Abs using 18 cm long gels was carried out. Figure 4 shows several typical examples of distribution by isoelectric points of the IgGs from the cerebrospinal fluids and sera of several patients. After isofocusing, the gels are stained with Coomassie blue. One can see that polyclonal CSF and serum IgGs are distributed over the entire isofocusing profile, but the IgGs staining intensity along the length of the gel can be different. For CSF IgG3 more intense staining is observed in the acidic pH zone, while for IgG8 in the alkaline zone, and IgG10 sub-fractions are located mainly in the neutral pH zone (Figure 4). It is interesting to see that the distribution of the CSF and sera IgGs from the same patient along the isoelectric points does not coincide, and the serum IgGs are more intensely distributed over the entire length of the gel. For mixtures of 10 IgG preparations from cerebrospinal fluid and serum, a more uniform distribution of antibodies between isoelectric points from 3 to 10 is observed (Figure 4). Seven individual preparations analyzed by us from cerebrospinal fluids and sera showed a variety of profiles of the distribution of antibodies at isoelectric points (Figure 4). This suggests that in the case of each MS patient, the synthesis of IgGs that differ in their properties may occur.

**Figure 5:**
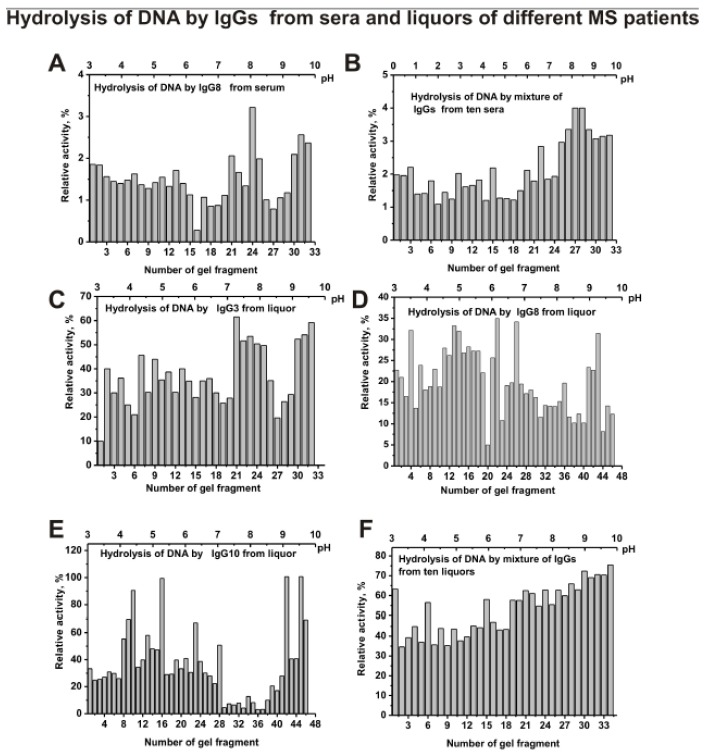
The patterns of the distribution of total IgGs with DNase activity corresponding to IgG8 from serum of individual MS patient (**A**) and s-IgG_mix_ (mixture of 10 IgG samples) (**B**) after their isofocusing. Similar analysis of the distribution of total polyclonal IgGs with DNase activity of individual CSFs (liquors) IgG3 (**C**), IgG8 (**D**), and IgG10 (**E**), as well as csf-IgG_mix_ (mixture of 10 IgG samples) (**F**). After cutting the gels into many fragments, the IgGs were extracted from the gel pieces using 30 µL of Tris-HCl buffer (pH 7.5) and 5 µL of the extracts was used for analysis of the relative activities in the hydrolysis scDNA; reaction mixtures were incubated for 17 h (**A**–**F**). The error in the initial rate calculation from three independent experiments did not exceed 5–10%.

After IgGs isofocusing, the gels were cut into 0.4-0.5 cm fragments. The extracts of the same or parallel gels were used for estimation of the relative activities of IgG’s sub-fractions separated by isoelectrofocusing in the hydrolysis of DNA, MBP, and five histones. Figure 5 demonstrates relative activity in the hydrolysis of scDNA by separated sub-fraction of individual IgG8 (A) and s-IgG_mix_—mixture of IgGs preparations from 10 sera (B), as well as IgG3 (C), IgG8 (D), IgG10 (E), and csf-IgG_mix_—mixture of IgGs preparations from 10 CSFs (F). The distribution of IgGs according to their isoelectric points (*pIs*) was very different for individual Ab preparations. The subfractions with specific *pIs* demonstrated different levels of DNase activity is (Figure 5A). However, in the case of the mixture of ten preparations of IgGs from different patients, an almost uniform distribution is observed over the entire isofocusing profile with a slight increase in activity in the pH range from 8 to 10 (Figure 5B).

Isofocusing separated sub-fractions of individual IgG3, IgG8, and IgG10 from CSFs demonstrating various relative activities in DNA hydrolysis significantly differed in their distribution at isoelectric points (Figure 5C–E). Similar data were obtained for four other individual CSF IgGs analyzed (data not shown). Interestingly, all subfractions of csf-IgG_mix_ (mixture of 10 preparations) located from pH 3 to 10 hydrolyze DNA with comparable efficiency (Figure 5F).

**Figure 6:**
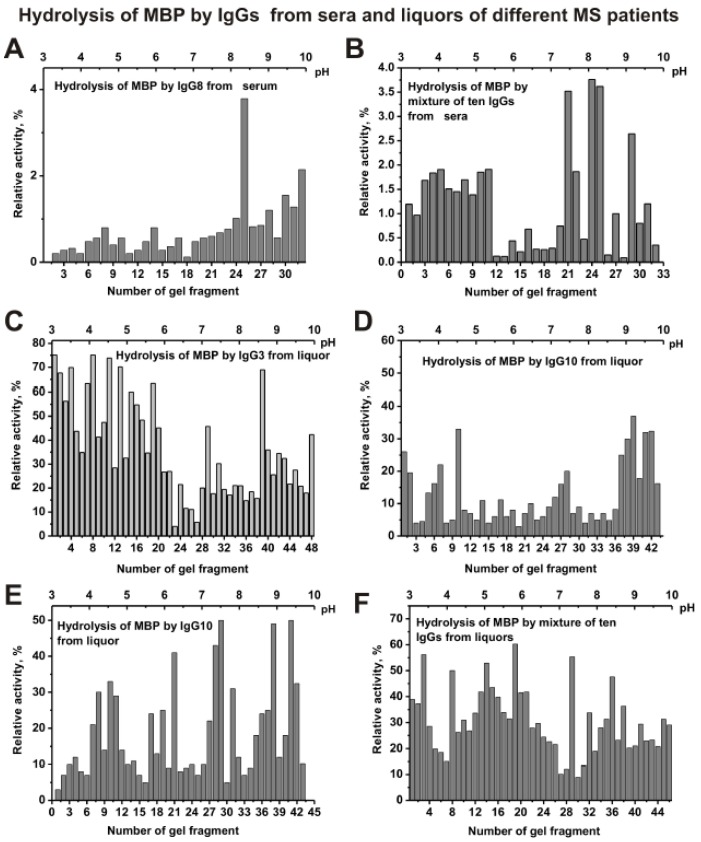
The patterns of the distribution of total IgGs with MBP-hydrolyzing activity corresponding to IgG8 from serum of individual MS patient (**A**) and s-IgG_mix_ (mixture of 10 IgG samples) (**B**) after their isofocusing. Similar analysis of the distribution of total IgGs with MBP-hydrolyzing activity of individual CSFs (liquors) IgG3 (**C**), IgG8 (**D**), and IgG10 (**E**), as well as csf-IgG_mix_ (mixture of 10 IgG samples) (**F**). After cutting the gels into many fragments, the IgGs were extracted from the gel pieces using 30 µL of Tris-HCl buffer (pH 7.5) and 5 µL of the extracts was used for analysis of the relative activities in the hydrolysis of MBP; reaction mixtures were incubated for 17 h (**A**–**F**). The error in the initial rate calculation from three independent experiments did not exceed 7–10%.

In the case of IgG8 from serum, the maximum activity in the hydrolysis of MBP is observed for its sub-fractions with isoelectric points from 8 to 10 (Figure 6A). However, s-IgG_mix_ demonstrates high activity at low pI (pH 3–5) and at high *pI* (pH 7.5–10) (Figure 6B). Sub-fractions of other individual serum IgGs with increased MBP-hydrolyzing activity were also corresponded mainly to the acidic (pH 3–5) and alkaline *pI* zones (pH = 8–10) of the gels (data not shown).

The distribution of IgG3 (Figure 6C), IgG8 (Figure 6D), IgG10 (Figure 6E), and other individual IgGs from CSFs (liquors) of MS patients by isoelectric points was very different. Nevertheless, csf-IgG_mix_ (mixture of ten samples) demonstrates many sub-fractions of IgGs with increased activity in the hydrolysis of MBP mainly in the *pI* zone from 4.5 to 6.5 (Figure 6F).

**Figure 7:**
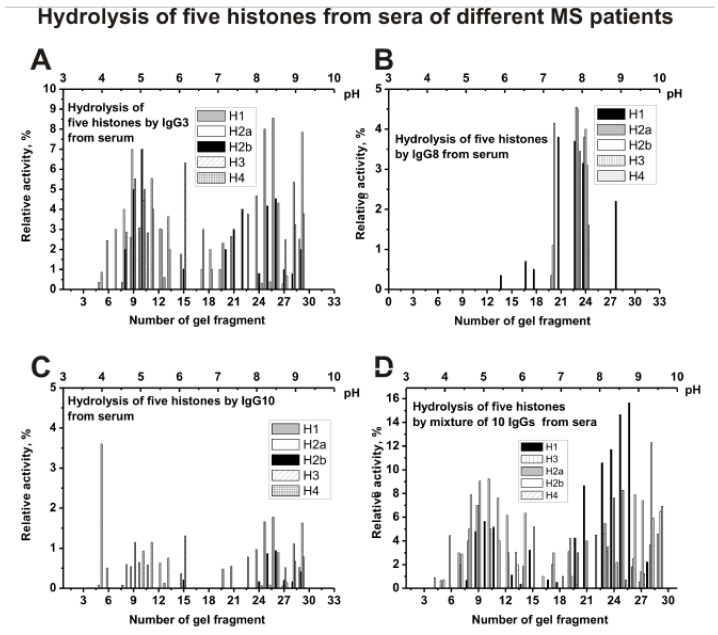
The patterns of the distribution of total IgGs with histone-hydrolyzing activity corresponding to serum IgG3 (**A**), IgG8 (**B**), and IgG10 (**C**), as well as s-IgG_mix_ (mixture of 10 IgG samples) (**D**) after their isofocusing. After cutting the gels into many fragments, the IgGs were extracted from the gel pieces using 30 µL of Tris-HCl buffer (pH 7.5) and 10 µL of the extracts were used for analysis of the relative activities in the hydrolysis of histones; reaction mixtures were incubated for 25 h (**A**–**D**). The error in the initial rate calculation from tree independent experiments depending of histone varied from 7 to 17%.

Figure 7 shows the distribution of individual IgG3 (A), IgG8 (B), IgG10 (C), and mixture of 10 serum IgGs (s-IgG_mix_) in the hydrolysis of five histones. The distribution of abzymes by isoelectric points for IgG8 is very different from other individual Ab preparations. IgG8 contains mainly subfractions that hydrolyze all five histones only in the *pI* zone from 7 to 8 (Figure 7B). For serum IgG3 (Figure 7A), IgG10 (Figure 7C), and other four analyzed individual IgGs, the distribution along the profile of histone-hydrolyzing activity is to some extent comparable to that for s-IgG_mix_ (Figure 7D).

**Figure 8:**
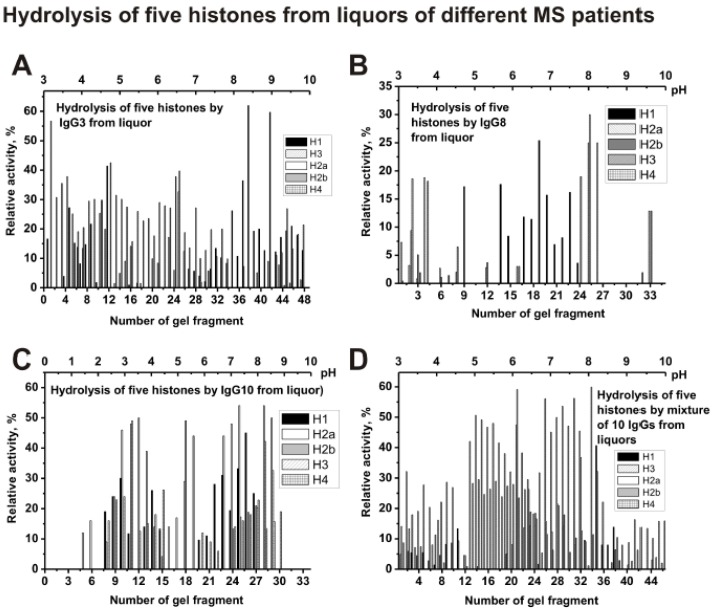
The patterns of the distribution of total IgGs with histone-hydrolyzing activity corresponding to CSFs (liquors) IgG3 (**A**), IgG8 (**B**), and IgG10 (**C**), as well as csf-IgG_mix_ (mixture of 10 IgG samples) (**D**) after their isofocusing. After cutting the gels into many fragments, the IgGs were extracted from the gel pieces and 1 µL of the extracts were used for analysis of the relative activities in the hydrolysis of histones; reaction mixtures were incubated for 17 h (**A**–**D**). The error in the initial rate calculation from three independent experiments depending of histone varied from 7 to 17%.

As one can see from Figure 8A that polyclonal IgG3 from CSF contain many Ab sub-fractions hydrolyzing all five histones with approximately comparable activities, which are distributed throughout the isofocusing profile. Significantly less sub-fractions of abzymes with proteolytic activity were revealed in the case of cerebrospinal fluid (liquor) IgG8 (Figure 8B) compared to IgG10 (Figure 8C) and other individual IgGs. For a mixture of Abs (csf-IgG_mix_), the distribution of abzymes along the profile (Figure 8D) is to some extent similar to that for an individual IgG3 (Figure 8A).

Thus, both the sera and CSFs IgGs of patients with MS can contain many abzymes with a variety of isoelectric points, which have DNase, MBP-, and histone-hydrolyzing activities. The isofocusing profiles DNase, MBP-, and histone-hydrolyzing activities may be very different for various individuals. Moreover, such profiles in the case of DNA-, MBP-, and histone-hydrolyzing activities of IgGs from the sera and CSFs (liquors) of the same patient do not match and may be very different. At the same time, mixtures of polyclonal IgGs from different MS patients with three enzymatic activities are distributed more evenly from pH 3 to 10 (Figure 5, Figure 6, Figure 7 and Figure 8).

## 4. Discussion

The presence of IgGs against DNA in CSF of patients with MS is in agreement with the detection of B-cells synthesizing anti-DNA Abs directly in active plaques and periplaque regions in MS brain and cerebrospinal fluids [2]. The presence of IgGs in CSFs of MS patients hydrolyzing DNA, MBP, and oligosaccharides was shown earlier [23,24,25]. In this paper, for the first time, evidence has shown that the CSF IgGs of patients with MS hydrolyze from one to five histones (H1, H2a, H2b, H3, and H4). The number and type of histones (H1, H2a, H2b, H3, and H4) hydrolyzing by polyclonal IgGs depends on the patient (Table 2). It was previously shown that auto-Abs and abzymes against DNA and MBP from sera of patients with SLE are very different in their affinity for these antigens [10,11,12,13,14,15,20,35,36]. In this work, the first analysis of the heterogeneity of IgGs from the cerebrospinal fluid was carried out. Figure 3 demonstrates significant distribution of CSF IgGs according to their affinity for three different ligands during Abs chromatographies on sorbents with immobilized DNA, MBP, and histones. Pictures of distribution by isoelectric points of IgGs from different patients can differ significantly (Figure 4, Figure 5, Figure 6, Figure 7 and Figure 8). However, the distribution of specific abzymes hydrolyzing DNA, MBP, and histones on the whole in the case of IgG mixtures occurs over the entire isofocusing profile from *pI* 3 to 10. It should be noted that some monoclonal IgGs of their total pool may have nearly the same affinity for each of these three antigens used, as well as have close *pI* values. Therefore, the analysis of their heterogeneity in terms of affinity for immobilized substrates and the relative activity in their hydrolysis can be significantly underestimated. Therefore, it is useful to note the data of some publications [37,38,39].

The cDNA library of only kappa light chains of Abs from patients with SLE was used to obtain monoclonal light chains (MLChs) ([37,38,39] and references therein). Only 45 of 451 and 33 of 687 individual colonies corresponding to one of 10 peaks eluted from DNA cellulose with 0.5 M NaCl and the second peak eluted with an acidic buffer (pH 2.6), respectively, were analyzed. Finally, 24 of 76 homogeneous MLChs (~32%), demonstrated DNase activity [37,38]. To analyze MLChs hydrolyzing MBP, 72 of 440 individual colonies were used, corresponding to one of 10 peaks eluted from the MBP-Sepharose with 0.5 M NaCl [39]. Of the analyzed 72 MLChs 25 (~35%) hydrolyzed MBP. All monoclonal abzymes against DNA and MBP showed different affinity for antigens, pH optima, dependence on various metal ions, etc. If take into account the average percentage of active monoclonal abzymes (~32–35%) in each of 10 eluted peaks containing abzymes, a possible number of monoclonal abzymes with DNase and MBP-hydrolyzing activity in total pool of SLE MLChs can be ≥1000 [40]. Taking into account extreme diversity and exceptional heterogeneity of abzymes, each MS stage may be accompanied by production of many different monoclonal auto-Abs without and with very different relative catalytic activities. In addition, abzymes of CSFs and sera of MS patients may be much more active in the hydrolysis of DNA, MBP, and histones than we have found (Table 1; Table 2), since the specific activity of IgGs was estimated using their total concentrations. It is important that the content of abzymes in sera of autoimmune patients should not exceed 1–10% of total immunoglobulins [10,11,12,13,14,15].

According to modern point of view, AI pathogenesis of various AIDs including MS and SLE agents as measles, hepatitis B, herpes simplex, influenza, papilloma, Epstein–Barr viruses, and different parasites may be involved [41,42,43]. Immune system can first produce Abs against viral or parasites proteins, and then it may switch to synthesis of auto-Abs to host antigens due to molecular mimicry between human and viral or bacterial proteins, alteration of host antigens, abnormal expression of immunoregulatory molecules, and activation of the anti-idiotypic network. Therefore, Abs can first be produced the components of viruses and bacteria and then to human antigens.

## 5. Conclusions

In this work, for the first time, the exceptional heterogeneity of IgGs of cerebrospinal fluid and serum of patients with MS in their affinity for DNA, MBP, and histones was demonstrated. It has been shown in this article that the abzymes of the CSF and sera IgG pools are very different in their isoelectric points and the relative catalytic activity in the hydrolysis of DNA, MBP, and histones.

## Figures and Tables

**Table 1:** Relative DNase activity of total proteins and IgGs from cerebrospinal fluid (CSF) and sera of patients with multiple sclerosis (MS) *.

№ of Patient	Relative Specific DNase Activity of IgGs; pmole DNA/1 mg of Ab/1 h		Relative Specific MBP-Hydrolyzing Activity, nmole MBP/1 h/mg		№ of Patient	Relative Specific DNase Activity of IgGs; pmole DNA/1 mg of Ab/1 h		Relative Specific MBP-Hydrolyzing Activity, nmole MBP/1 h/mg	
	CSF	Serum	CSF	Serum		CSF	Serum	CSF	Serum
1	440.8	11.7	136	0.8	15	460.6	12.9	334.7	3.4
2	596.7	3.5	18.8	0.24	16	83.3	7	160.0	1
3	649.1	2.4	75.4	0.95	17	250	12.1	104.5	0.4
4	416.7	16	72.2	2.5	18	590.7	14.4	118.7	0.9
5	470.5	7	3.2	0.21	19	654.1	14.7	161.1	2.8
6	452.5	7.5	40.8	6.7	20	316.7	8.3	125.5	0.2
7	453.5	8.7	104.6	2.9	21	370.5	10.1	201.2	6.9
8	107.75	11.7	219.7	2.7	22	353.5	12.1	308.4	3
9	97.8	9.6	251.1	3.3	23	253.5	9.7	251.1	2.8
10	175.8	5.2	125.5	2.8	24	792.1	9.9	242.1	3.7
11	992.1	13.9	209.2	6.5	25	793.8	15.2	138.7	3.5
12	773.8	8.1	303.4	4.8	26	148.2	9.2	219.7	6.9
13	143.2	2.6	251.1	2.1	27	530.4	14.8	249.1	5
14	524.4	7.7	251.1		28	465.6	7.3	146.5	2.8
Average value **	-	-	-	-	-	440.5 ± 232.8	9.6 ± 4.0	172.3 ± 90.4	3.1 ± 2.2
Ratio	45.0		55.6			45.0		55.6	
CC	+0.26		+0.45			+0.26		+0.45	

* For each value, the mean of three measurements is reported; the error of the determination of values did not exceed 7–10%. ** Average values are given as mean ± S.D.

**Table 2:** Relative protease activity of IgGs from CSF and sera of patients with MS *.

№ of Patient	Relative Activity of the Hydrolysis, % **									
	H1		H2a		H2b		H3		H4	
	CSF	Serum	CSF	Serum	CSF	Serum	CSF	Serum	CSF	Serum
1	671	0	194	0	100	0	103	0	170	21
2	215	0	110	25	120	0	167	0	100	5
3	0	14.1	130	15	50	12	70	0	135	0
4	88	18	140	0	0	0	102	5	100	0
5	121	24	220	0	110	13.2	170	0	170	7
6	114	0	150	29	10	0	213	0	70	7
7	100	0	322	0	70	0	145	12	210	5
8	50	0	251	17	90	7	280	0	370	7
9	340	14	440	24	14	0	312	0	120	21
10	311	0.7	110	12	7	0	50	5	51	0
11	36	1.0	330	7	9	0	90	7	140	4
12	240	2.5	50	5	50	5	44	6	24	0
13	101	9.0	170	4	43	0	200	10	49	0
14	304	11.6	251	7.8	12	0	302	5	524	15
15	97	21.5	137	0	15	0	376	0	0	0
16	29	0.4	60	14.4	90	0	142	2.1	18	0
17	114	0	0	20.8	110	0	10	0	5	0
18	5	5	47	28.1	8	0	164	4	31	7.2
19	126	0	159	27	30	0	0	0	24	4.2
20	134	6	293	16.9	50	10	100	10	12.8	0
21	10	1.6	160	12.4	9	0	160	12	48	8.8
22	0	20	318	21	4	5	40	0	19	0
23	7	5	319	0	0	0	120	15	159	12
24	0	4	292	14	9	0	105	14	352	4.8
25	9	9	275	27	12	0	220	21	131	0
26	0	0	274	0	14	11	120	19	393	5
27	0	10	198	0	12	0	10	0	375	7
28	190	15	346	14	80	0	8	0	310	9
Average value	121.9 ± 148.6	6.9 ± 7.6	205.2 ± 107	12.3 ± 10.3	40.3 ± 39.6	2.2 ± 4.3	136.5 ± 98.3	5.3 ± 6.4	147 ± 143	5.4 ± 6.0
Difference	17.7		16.8		18.3		25.8		27.2	
Median	98.5	4.5	196	13.2	14.5	0	120	3.1	110	4.9
IQR	154	12.8	159	20.9	66	2.5	125	10	162	7.1
CCs	−0.1		−0.1		+0.23		+0.05		+0.43	
CC; with DNase	−0.22	0.05	−0.02	+0.03	−0.16	−0.3	−0.2	+0.56	+0.05	+0.05
CC; with MBP-hydrolysis	−0.14	−0.13	+0.28	−0.15	−0.46	−0.18	+0.18	+0.32	+0.16	+0.19

* For each value, a mean of three independent measurements is reported; the error of the determination of every of three values used did not exceed 7%-15%. **The reaction mixtures for analysis of histone-hydrolyzing activities of IgGs 0.1–0.2 mg of IgGs in the case of sera IgGs and 0.005–0.03 mg/mL IgGs from CSFs. They were incubated for 3–15 h. The data obtained then were recalculated to standard conditions: percent of the hydrolysis in the presence of 0.1 mg/mL IgGs during 15 h.

## Supplementary Materials

The following are available online at https://www.mdpi.com/2218-273X/10/4/630/s1, Table S1: Several different characteristics of MS patients.

## Abbreviations

Abs	Antibodies, abzymes, catalytically active antibpdies
AIDs	Autoimmune diseases
BSA	Bovine serum albumin
CC	Correlation coefficient
CSF	Cerebrospinal fluid
DTT	dithiothreitol
FPLC	Fast protein liquid chromatography
IEF	isoelectrofocusing
MBP	myelin basic protein
MS	multiple sclerosis
MLChs	monoclonal light chains
RA	relative activity
SDS-PAGE	SDS-polyacrylamide gel electrophoresis
SLE	systemic lupus erythematosus
scDNA	Supercoiled DNA
relDNA	relaxed DNA
