# Supplementary material for: Extreme Diversity of IgGs Against Histones, DNA, and Myelin Basic Protein in the Cerebrospinal Fluid and Blood of Patients with Multiple Sclerosis
Source: Biomolecules. 2020 Apr 18;10(4):630. doi: 10.3390/biom10040630 (PMC7226582; doi:10.3390/biom10040630)
Supplement: Supplementary file 1 [file biomolecules-10-00630-s001.pdf]

Supplementary Table S1. Several different characteristics of MS patients

№	Sex	Age (years)	MS subtypes	Clinical activity*	MRI activity $^{\xi}$	Disease Duration (months)	EDSS$^{\beta}$	IgG CSF (mg/dL)	IgG serum (mg/dL	IgG oligoclonal bands (OCB)
1	F	59	RR**	YES	NO	4	3.0	7.5	1660	Identical bands in CSF and in serum
2	F	28	RR	NO	NO	2	3.0	3.4	1010	OCB only in CSF
3	M	36	RR	YES	NO	1	1.5	5.0	995	OCB only in CSF
4	F	26	RR	NO	NO	5	3.0	1.9	1184	OCB only in CSF
5	M	49	RR	YES	YES	24	4.0	6.0	1189	OCB only in CSF
6	F	20	RR	YES	YES	18	3.0	13.6	1450	OCB only in CSF
7	M	46	PP*	NO	NO	3	3.0	6.4	1060	OCB only in CSF
8	M	51	PP	YES	YES	10	3.5	11.4	1190	OCB only in CSF
9	F	31	RR	YES	YES	2	2.5	2.2	787	No OCB bands
10	F	26	RR	YES	YES	48	2.0	9.0	1260	OCB only in CSF
11	F	43	RR	YES	YES	12	4.0	3.2	1290	OCB only in CSF
12	F	45	RR	YES	YES	15	3.0	2.4	1490	No OCB bands
13	M	30	PP	YES	YES	14	3.0	7.0	1160	OCB only in CSF
14	F	60	PP	YES	NO	36	2.0	11.3	1080	OCB only in CSF
15	F	29	RR	NO	YES	7.2	3.0	2.5	1000	OCB only in CSF
16	F	41	RR	YES	NO	5	2.5	4.6	1140	No OCB bands
17	M	43	RR	YES	YES	3	0.5	5.5	1070	OCB only in CSF
18	F	44	RR	YES	NO	1	1.0	5.5	1082	OCB only in CSF
19	F	21	RR	YES	YES	3	2.5	3.7	883	OCB only in CSF
20	F	21	RR	YES	YES	3	2.5	3.7	883	OCB only in CSF
21	M	32	RR	NO	NO	6	1.0	2.56	597	OCB only in CSF
22	F	40	RR	YES	YES	7	6.5	5.5	1076	OCB only in CSF
23	F	34	RR	NO	NO	1	1.0	4.36	1030	OCB only in CSF
24	F	52	RR	NO	NO	11	1.5	9.08	1080	OCB only in CSF
25	F	58	PP	NO	NO	24	4.5	5.1	961	OCB only in CSF
26	F	52	RR	YES	NO	4	3.5	2.71	810	OCB only in CSF
27	M	45	RR	NO	YES	3	0	5.9	844	OCB only in CSF
28	F	21	RR	YES	YES	3	2.5	3.7	883	OCB only in CSF
Average values	20 F 8 M	38.7± 12.3	23 RR 5 PP	19 YES 9 NO	15 YES 13 NO	10.8± 11.3	2.6± 1.3	5.5±3.0	1076.6± 224.6	25 Yes and 3 No

*Clinical activity corresponds to the presence of relapse at the time of sampling

**Relapsing–remitting (RR) and primary progressive (PP) MS

$^{\xi}$MRI activity = presence or absence gadolinium enhancing lesions at MRI examination

$^{\beta}$Kurtzke's expanded disability status scale (EDSS)
